# Prospective Evaluation of ResistancePlus MG, a New Multiplex Quantitative PCR Assay for Detection of Mycoplasma genitalium and Macrolide Resistance

**DOI:** 10.1128/JCM.02312-16

**Published:** 2017-05-23

**Authors:** S. N. Tabrizi, J. Su, C. S. Bradshaw, C. K. Fairley, S. Walker, L. Y. Tan, E. Mokany, S. M. Garland

**Affiliations:** a Department of Microbiology and Infectious Diseases, The Royal Women's Hospital, Parkville, Victoria, Australia; b Department of Obstetrics and Gynaecology, University of Melbourne, Victoria, Australia; c Murdoch Childrens Research Institute, Parkville, Victoria, Australia; d Melbourne Sexual Health Centre, Carlton, Victoria, Australia; e Central Clinical School, Monash University, Frankston, Victoria, Australia; f SpeeDx Pty Ltd., Sydney, NSW, Australia; Cleveland Clinic

**Keywords:** Mycoplasma genitalium, macrolide resistance, 23S rRNA, multiplex assay, NAAT, qPCR, diagnostic, sensitivity and specificity

## Abstract

Mycoplasma genitalium is a significant pathogen for which first-line treatment is becoming less effective due to increased resistance to macrolides. As conventional culture and antimicrobial susceptibility testing is not feasible for routine detection of this pathogen, molecular markers such as detection of mutations in the 23S rRNA gene have been described to predict resistance. Recently, a novel multiplex quantitative PCR (qPCR) assay, ResistancePlus MG, has been described for the simultaneous detection of Mycoplasma genitalium and macrolide resistance. In the current study, the clinical performance of the assay was evaluated on 1,089 consecutive urine and anogenital swab samples in symptomatic and asymptomatic male and female patients. Overall, 6.0% were positive for M. genitalium, with 63.1% having macrolide resistance-associated mutations. Compared to the laboratory-validated qPCR method targeting the 16S rRNA gene and Sanger sequencing to determine 23S rRNA mutations, the sensitivity and specificity of M. genitalium detection were 98.5% and 100% and for detection of macrolide resistance mutations were 100.0% and 96.2%, respectively. This assay offers a considerable advantage in clinical settings for M. genitalium testing by making the results of macrolide resistance and mutation analyses simultaneously available, which is increasingly important with escalating macrolide resistance.

## INTRODUCTION

Mycoplasma genitalium is a significant pathogen in the etiology of nongonococcal urethritis (NGU) in men (1, 2) and of cervicitis, pelvic inflammatory disease (PID), and infertility in women (3–7). Infections and syndromes associated with M. genitalium such as NGU are often treated with the macrolide antibiotic azithromycin (8, 9). However, over the past decade, there have been increasing reports of declining cure rates, which in some settings are now as low as 50% in genital M. genitalium infections in symptomatic men and women (10–13). Azithromycin treatment failure is strongly associated with the presence of mutations in binding region V of the M. genitalium 23S rRNA gene (equivalent to Escherichia coli nucleotide positions 2058 and 2059) (11, 14).

M. genitalium is a fastidious, slow-growing organism and is challenging to culture. Therefore, traditional culture-based methods are not possible for routine diagnosis; hence, detection requires nucleic acid amplification tests (NAATs). Conventional culture-based antibiotic susceptibility tests are therefore also not feasible. However, prediction of resistance can be inferred from the detection of the macrolide resistance-mediating mutations in the 23S rRNA gene (13, 14).

Different NAATs have been utilized for the detection of macrolide resistance mutations. DNA sequencing has been used to detect mutations in cases of treatment failure and is also the gold standard method for mutant determination (14). Sequencing, however, is generally not feasible for routine diagnosis due to the higher cost and longer turnaround time to results, delaying the reporting of macrolide resistance and hence treatment with second-line antimicrobials. Alternatively, other real-time quantitative PCR (qPCR)-based approaches have been utilized such as high-resolution melt assay (HRMA)-based methods (15, 16), a 5′ nuclease assay, and the use of mutation-specific primers (17, 18). These methods were limited in their ability to detect the macrolide resistance mutations in all M. genitalium-positive samples or were restricted in sensitivity and the ability to multiplex. These methods have been demonstrated only on M. genitalium-positive samples, with the exception of the fluorescence resonance energy transfer (FRET) method; however, this was limited in sensitivity for M. genitalium detection (19).

ResistancePlus MG is a new multiplex qPCR assay employing novel PlexZyme and PlexPrime technology (SpeeDx Pty Ltd., Sydney, Australia) that has recently been developed (20) and that allows simultaneous detection of M. genitalium and the common mutations associated with macrolide resistance (including A2058G, A2059G, A2058T, A2058C, and A2059C) in one well (21). The previous evaluation assessed clinical performance in an infected population and also analytical performance (21). In this study, a prospective clinical evaluation of this assay was conducted in a routine clinical setting, thereby reflecting the prevalence of M. genitalium and mutations associated with macrolide resistance in this population. The performance of the assay for both M. genitalium detection and determination of mutation status was compared to that of a laboratory-validated qPCR method targeting the 16S rRNA gene and Sanger sequencing of the 23S rRNA gene.

## RESULTS AND DISCUSSION

The specimens included 388 samples comprising 354 urine/urethral swabs and 34 anal swabs from men and 701 samples comprising 203 urine and 497 cervical/vaginal swabs and 1 rectal swab from women (Table 1).

**TABLE 1:** Specimen types and reference assay results

Assay result	No. (%) of specimens						
	Male			Female			Total
	Urine/urethral	Anal swab	All	Urine	Vaginal/cervical/rectal swab	All	
M. genitalium							
Detected	35 (9.9)	7 (20.6)	42 (10.8)	14 (6.9)	9^*a*^ (1.8)	23 (3.3)	65 (6.0)
Not detected	319 (90.1)	27 (79.4)	346 (89.2)	189 (93.1)	489 (98.2)	678 (96.7)	1,024 (94.0)
Total	354 (100)	34 (100)	388 (100)	203 (100)	498 (100)	701 (100)	1,089 (100)
23S rRNA status							
Wild type	8 (22.9)	0 (0)	8 (19.0)	12 (85.7)	4 (44.4)	16 (69.6)	24 (36.9)
Mutant^*b*^	27 (77.1)	7 (100)	34 (81.0)	2 (14.3)	5^*a*^ (55.6)	7 (30.4)	41 (63.1)
Total	35 (100)	7 (100)	42 (100)	14 (100)	9 (100)	23 (100)	65 (100)

a Includes one rectal sample from a female.

b Mutations included 23 A2058G, 13 A2059G, 4 A2058T, and 1 A2058C.

Overall, 6.0% (65/1,089) of the samples were positive for M. genitalium by the reference 16S rRNA gene assay, with 10.8% of male and 3.3% of female samples being positive. A 23S rRNA mutation was detected in 63.1% (41/65) of the positive samples by Sanger sequencing (Table 1). A total of 34 (81.0%) samples from males had macrolide resistance mutations compared to 7 (30.4%) samples from females (*P* < 0.0001). Overall, the prevalence of M. genitalium and macrolide-resistant mutations was significantly higher in men (*P* < 0.0001), as they were primarily from symptomatic patients at the Melbourne Sexual Health Centre (MSHC), Victoria, Australia. It is noteworthy that macrolide resistance mutations were detected in all M. genitalium-positive rectal swabs, highlighting a particularly challenging issue in treatment of rectal infections.

The level of concordance of the ResistancePlus MG assay with the reference method for detection of M. genitalium was 99.9%, with a Kappa value of 0.99 (95% confidence interval [CI], 0.98 to 1.00) and sensitivity and specificity of 98.5% and 100.0%, respectively. In this routine clinical setting, the positive and negative predictive values for detection of M. genitalium were 100% and 99.9% and the positive and negative predictive values for detection of 23S rRNA mutations were 97.4% and 100%, respectively (Table 2). Overall, only 1 discordant M. genitalium result was obtained: a positive female urine sample referred from an external laboratory, which had a M. genitalium load of less than 100 copies in the sample. This was detected by the reference assay; however, the value was near the cutoff, and the ResistancePlus MG assay returned a negative result. When this sample was retested in duplicate by the reference method, it resulted in a negative result and a positive result reflective of a sample with low bacterial load and at the threshold of the detection limit.

**TABLE 2:** Evaluation of the ResistancePlus MG assay for the detection of M. genitalium and 23S rRNA gene mutations^*a*^

Parameter	ResistancePlus MG result	Reference assay result (no. of specimens)^*b*^			% sensitivity (95% CI)/% specificity (95% CI) (negative predictive value [95% CI]/positive predictive value [95% CI])
		Pos	Neg	Total	
M. genitalium	Pos	64	0	64	98.5 (91.7–99.9)/100 (99.6–100) (99.9 [99.5–100]/100 [94.4–100])
	Neg	1^*c*^	1,024	1,025
23S rRNA mutation	Detected	38	1	39	100 (90.8–100)/96.2 (80.4–99.9) (100 [86.3–100]/97.4 [86.5–99.9])
	Not detected	0	25	25

a Pos, positive; Neg, negative.

b The reference assay used for M. genitalium detection was a standard assay in use in the laboratory (22, 23), and the assays used for 23S rRNA mutation status detection were Sanger sequencing and HRMA (14, 16). In the comparison of mutation detection results, Pos by the reference assay represents detection of a mutant strain and Neg represents detection of the wild-type strain.

c Fewer than 100 copies of M. genitalium were detected in the sample.

Among the 64 specimens positive for M. genitalium by the ResistancePlus MG assay, 63 (98.4%) were concordant with sequencing for 23S rRNA mutant detection, with a Kappa value of 0.97 (95% CI, 0.90 to 1.00) and sensitivity and specificity of 100% and 96.2%, respectively (Table 2).

Overall, 88% of the mutations represented in this cohort, as detected by Sanger sequencing, were A2058G or A2059G. Among the 5 mutation types detectable by the ResistancePlus MG assay, all except A2059C were present in this patient population and results corresponded to the Sanger sequencing result. Previously, it was shown that all 5 mutations are detectable with similar analytical sensitivities (21); however, detection of all these mutations and the clinical performance of the analyses would need to be determined with further studies and also in other populations. This study did not collect detailed symptoms from each patient, and, as such, further studies would need to be performed to evaluate the utility of M. genitalium and macrolide testing in asymptomatic patients. The data presented indicate that 63.1% of the specimens had macrolide resistance-associated mutations; however, these single nucleotide mutations confer high-level resistance to azithromycin and have been consistently associated with clinical failure of this drug (10, 24). Comparison to phenotypic resistance assay results was not possible in this study due to the well-established difficulty in culturing M. genitalium; however, phenotypic data have been correlated with resistance mutations (14).

The ResistancePlus MG assay performed extremely well against the reference methods utilized in our laboratory, and, unlike the sequencing method, this assay does not require specialized instrumentation and can be performed by routine diagnostic laboratories. It also offers the unique capability of simultaneous M. genitalium detection and assessment of macrolide resistance. Patients infected with M. genitalium 23S rRNA mutants are predicted to fail treatment. This study aimed to evaluate the assay with consecutive samples sent to the laboratory and included a mixture of specimens from symptomatic and asymptomatic patients that had been sent to the laboratory for testing. Although the details of the individual patient symptoms were not available, the alarmingly higher prevalence of M. genitalium and resistance mutations detected in the male population, which consisted primarily of men from a sexual health clinic who were symptomatic with urethritis, highlights the importance of utilizing this assay for detection of M. genitalium and resistance testing to inform management of disease. This assay offers a considerable advantage for the rapid detection of macrolide-resistant strains and can be incorporated into diagnostic algorithms which can individualize antimicrobial therapy to ensure rapid delivery of agents to which the organism is susceptible. Where azithromycin is used as the first-line therapy, simultaneous availability of M. genitalium detection and detection of macrolide resistance mutations would allow clinicians to rapidly recall patients to provide a more appropriate second-line treatment in comparison to waiting for up to 4 weeks for test of cure or treatment failure. Where doxycycline rather than azithromycin is used as first-line therapy for sexually transmitted infection (STI) syndromes, patients with M. genitalium infection can be recalled for treatment with an antimicrobial based on the macrolide resistance profile. Macrolide-susceptible strains can be treated with azithromycin and macrolide-resistant strains with a quinolone such as moxifloxacin. Future assays that provide quinolone resistance data would assist in further refining clinical algorithms by identifying patients with dual-class resistance. Of note, 9% of M. genitalium strains at Melbourne Sexual Health Centre in Melbourne, Australia, currently have dual-class resistance (macrolide and quinolone), resulting in the need to use antimicrobials such as pristinamycin which have very limited availability outside Europe (25). Clinical algorithms such as this that incorporate resistance testing and individualize care have the potential to greatly improve microbial cure, to promote antimicrobial stewardship, and to reduce clinic visits and, ultimately, the spread of antibiotic-resistant bacteria in populations.

## MATERIALS AND METHODS

### Patient populations and sample types.

Overall, 1,089 samples consecutively received over the course of 2 months (November to December 2015) were utilized for this evaluation. Specimens included 469 (344 from men and 125 from women) from the Melbourne Sexual Health Centre (MSHC), Victoria, Australia; 511 (12 from men and 499 from women) from the Royal Women's Hospital (RWH), Victoria, Australia; and 109 (32 from men and 77 from women) referred from external laboratories. The patient population from MSHC consisted primarily of symptomatic patients with NGU, cervicitis, and proctitis and/or PID, as well as of sexual contacts of M. genitalium-infected partners. The RWH samples were primarily from nonsymptomatic female patients presenting for contraceptive advice or for insertion of an intrauterine contraceptive device or referrals for medical and/or surgical termination of pregnancy, plus a small number of male partners referred for testing. Other samples were referred by external laboratories for diagnostic testing. Ethical approval for this study was granted by the Royal Women's Hospital Research Ethics Committee.

### Sample processing, amplification, sequencing, and detection.

Urine and flocked swab (Copan Diagnostic, Brescia, Italy) samples were processed and specimens extracted as described previously (10). Detection of M. genitalium by the ResistancePlus MG assay was performed in a blind manner with respect to the other results, and the data obtained were compared to the results of the laboratory-validated qPCR method targeting the 16S rRNA gene. Detection of macrolide resistance by the ResistancePlus MG assay was compared to detection of 23S rRNA mutations using Sanger sequencing (14). The ResistancePlus MG assay was performed simultaneously with the same extracted nucleic acid as was utilized for the reference method, according to the manufacturer's protocol (using the Beta version as previously described [21]). Briefly, an aliquot of 5 μl extracted DNA (equivalent to 50 μl of urine and 1/40 of the total swab specimen) was amplified for detection in a 20-μl reaction volume; targets are selected across three fluorescent channels on a LightCycler 480 II real-time instrument (Roche Diagnostic, Indianapolis, IN, USA) for M. genitalium detection using the MgPa gene, 5 stacked 23S rRNA mutations, including A2058G, A2059G, A2058T, A2058C, and A2059C, and an internal control target; and data were analyzed using an analysis algorithm provided with the assay, as described previously (21).

Fisher's exact test was utilized to compare the data with respect to positivity and resistance between variables.
